# Perfluoroalkanesulfonamide Organocatalysts for Asymmetric Conjugate Additions of Branched Aldehydes to Vinyl Sulfones

**DOI:** 10.3390/molecules181214529

**Published:** 2013-11-25

**Authors:** Kosuke Nakashima, Miho Murahashi, Hiroki Yuasa, Mariko Ina, Norihiro Tada, Akichika Itoh, Shin-ichi Hirashima, Yuji Koseki, Tsuyoshi Miura

**Affiliations:** 1 Tokyo University of Pharmacy and Life Sciences, 1432-1 Horinouchi, Hachioji, Tokyo 192-0392, Japan; 2 Gifu Pharmaceutical University 1-25-4, Daigaku-nishi, Gifu 501-1196, Japan

**Keywords:** organocatalyst, sulfonamide, vinyl sulfone, conjugate addition, quaternary stereocenters, fluorous

## Abstract

Asymmetric conjugate additions of branched aldehydes to vinyl sulfones promoted by sulfonamide organocatalyst **6** or **7** have been developed, allowing facile synthesis of the corresponding adducts with all-carbon quaternary stereocenters in excellent yields with up to 95% ee.

## 1. Introduction

All-carbon quaternary stereocenters are one of the most important motifs in many natural products and bioactive compounds; however, relatively harsh reaction conditions are required to construct these stereocenters due to their steric hindrance. In addition, combinations of electrophile and nucleophile are limited, and the stereoselective construction of all-carbon quaternary stereocenters is not generally straightforward. Therefore, development of efficient synthetic methods to stereoselectively construct all-carbon quaternary stereocenters under mild reaction conditions is highly desirable in organic synthesis [1,2,3,4,5,6,7,8,9,10,11,12,13]. Among various methodologies to construct these centers, organocatalysis is one of the most effective processes that can be performed under mild conditions. The synthetic methods for compounds with quaternary stereogenic centers using organocatalysts have received considerable attention, particularly in the field of green chemistry [14,15]. Michael additions of various carbonyl compounds to 1,1-bis(benzenesulfonyl)ethylene (**11**) using organocatalysts are efficient synthetic methods, and several research groups have reported findings in this area [16,17,18,19,20,21,22,23,24,25,26,27,28,29,30,31,32,33]; however, successful conjugate additions of α-branched aldehydes and **11** for the construction of such all-carbon quaternary stereocenters have been rarely reported [34,35,36,37,38]. Alexakis and coworkers reported that l-proline derivatives catalyze the reaction of **11** with α-branched aldehyde **12a** to give the corresponding adduct **13a** with up to 73% ee [34,35]. Lu and coworkers reported that the sulfonamide organocatalyst derived from l-threonine promotes the conjugate addition of **12a** to **11** in the unusual reaction solvent *p*-fluorotoluene to afford the corresponding adduct **13a** in high yield with high enantioselectivity (up to 83% ee) [36]. Furthermore, Maruoka and coworkers reported efficient conjugate additions of α-heterosubstituted aldehydes with **11** using a sulfonamide organocatalyst with a dihydroanthracene framework (up to 95% ee) [37]. Recently, we also reported that a diaminomethylenemalononitrile organocatalyst catalyzes similar conjugate additions to afford **13a** with high enantioselectivity (up to 89% ee) [38].

**Figure 1:**
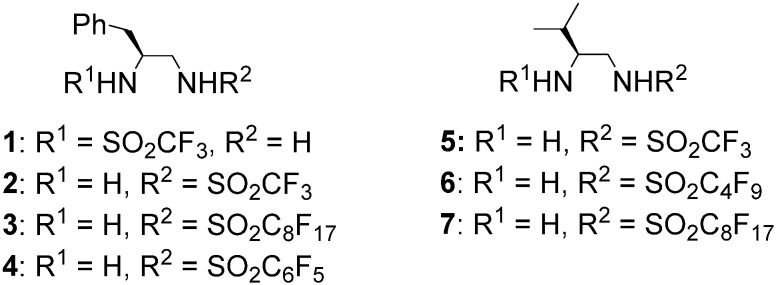
Structure of organocatalysts.

On the other hand, fluorous compounds with a perfluoroalkyl group can be easily separated from nonfluorous compounds by fluorous organic solvent extraction or fluorous solid phase extraction (FSPE) using fluorous silica gel [39]. Several research groups have reported asymmetric reactions in which fluorous organocatalysts are recyclable [40]. We have also reported a direct aldol reaction in water using fluorous sulfonamide organocatalyst **3** and related catalysts [41,42,43], Michael addition reactions using a fluorous thiourea organocatalyst [44], and an oxidation reaction using fluorous IBX [45]. In addition, we have reported a method for the synthesis of both enantiomeric aldol products in water using sulfonamide organocatalysts **1** [46] and **2** [47,48], prepared from l-phenylalanine. Very recently, we reported in a preliminary communication that perfluoroalkanesulfonamides **5** and **6** catalyze the conjugate additions of branched aldehydes to vinyl sulfone **11** to give the corresponding adducts with excellent stereoselectivities [49]; however, development of a protocol for recovery and reuse of **5** and **6** is yet to be reported. Herein, we describe the full details of the conjugate additions of branched aldehydes to vinyl sulfone using **6** and novel fluorous sulfonamide **7** (Figure 1).

## 2. Results and Discussion

**Scheme 1:**
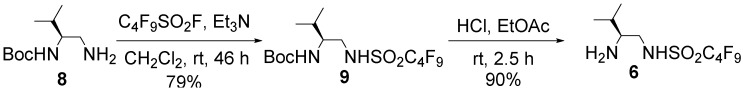
Preparation of organocatalyst **6**.

**Scheme 2:**
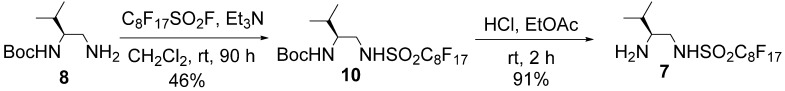
Preparation of organocatalyst **7**.

We initially examined the sulfonamide organocatalysts **1**–**7** for the conjugate addition of **12a** to **11** as a test reactant (Table 1). Sulfonamide organocatalysts **1**–**4** derived from l-phenylalanine were superior to catalyst **5** derived from l-valine for the direct aldol reactions in water [37,46,48]; however, **5** bearing the valine skeleton resulted more suitable for the conjugate addition with vinyl sulfone **11** (entries 1–5). Furthermore, to develop a more powerful organocatalyst, we synthesized **6**, which enhanced the acidity of the sulfonamide group by the introduction of the perfluorobutyl group. Treatment of compound **8** [50] with perfluorobutanesulfonyl fluoride in presence of triethylamine in dichloromethane provided the intermediate **9** in 79% yield. The Boc protective group was removed by treatment of hydrogen chloride in ethyl acetate to give the desired perfluorobutanesulfonamide **6** in 90% yield (Scheme 1). Organocatalyst **6** was more effective for conjugate additions with vinyl sulfone **11**, resulting in the highest enantioselectivity (91% ee) and excellent yield (entry 6). Furthermore, to develop an organocatalyst that can be recovered and reused, **7** was synthesized by a similar procedure (Scheme 2). The stereoselectivity was slightly reduced in the reaction using **7** (entry 7).

**Table 1:** Selection of organocatalyst. 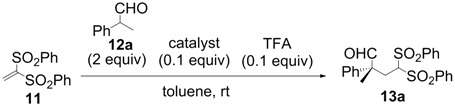

Entry	Catalyst	Time (h)	Yield ^a^ (%)	ee ^b^ (%)
1	**1**	1	95	−2
2	**2**	1.5	99	80
3	**3**	2	88	86
4	**4**	2	97	79
5	**5**	2	95	88
6	**6**	2	100	91
7	**7**	2	99	89

^a^ isolated yield; ^b^ determined by HPLC analysis.

We investigated the optimal reaction conditions for the enantioselective conjugate additions using **6**, various solvents, and additives (Table 2). Conjugate additions were performed with vinyl sulfone **11** and 2-methylphenylethanal (**12a**) as test reactants in the presence of a catalytic amount of **6** and trifluoroacetic acid (TFA) at room temperature. A slight reduction in enantioselectivity and much longer reaction time were observed without TFA (entries 1 and 2). Aprotic solvents such as dichloromethane, diethyl ether, ethyl acetate, acetonitrile, chloroform, 1,2-dichloroethane, and *p*, *m*, and *o*-xylene were accepted well in this conjugate addition with good enantioselectivity (entries 3 and 5–12). A protic polar solvent such as methanol is a poor solvent for this reaction and provided low yield and enantioselectivity (entry 4). Among the solvents probed, the best results (95% yield and 93% ee) were achieved when the reaction was performed in *m*-xylene (entry 11). We also examined the effects associated with the presence of other protic acids, including benzoic acid, *p*-nitrobenzoic acid, and trifluoromethanesulfonic acid; however, TFA was found to be the most suitable additive (entries 13–15). Additions of 0.2 or 0.05 equiv of TFA resulted in a slight reduction in stereoselectivity (entries 16 and 17). The highest enantioselectivity (95% ee) was obtained when the reaction was performed at 0 °C or −10 °C, although longer reaction time (21 h or 72 h) was required (entries 18 and 19). Enantioselectivity was slightly reduced when the catalyst loading was lowered to 0.05 equiv (entry 20). Considering the reaction time, the optimal conditions were determined to be 0.1 equiv of **6** and 0.1 equiv of TFA in *m*-xylene at room temperature (entry 11).

**Table 2:** Optimization of reaction conditions using organocatalyst **6**. 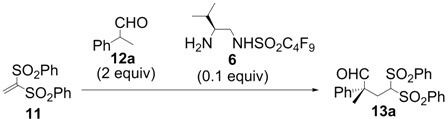

Entry	Solvent	Temp	Additive (equiv)	Time (h)	Yield ^a^ (%)	ee ^b^ (%)
1	toluene	rt	none	24	75	86
2	toluene	rt	TFA (0.1)	2	100	91
3	CH_2_Cl_2_	rt	TFA (0.1)	2.5	97	91
4	MeOH	rt	TFA (0.1)	24	33	32
5	Et_2_O	rt	TFA (0.1)	5	87	82
6	EtOAc	rt	TFA (0.1)	2.5	98	86
7	MeCN	rt	TFA (0.1)	4	87	77
8	CHCl_3_	rt	TFA (0.1)	2	99	91
9	ClCH_2_CH_2_Cl	rt	TFA (0.1)	2	98	92
10	*p*-xylene	rt	TFA (0.1)	2	94	92
11	*m*-xylene	rt	TFA (0.1)	2	95	93
12	*o*-xylene	rt	TFA (0.1)	2	97	89
13	*m*-xylene	rt	PhCO_2_H (0.1)	5.5	46	80
14	*m*-xylene	rt	4-NO_2_C_6_H_4_CO_2_H (0.1)	24	85	82
15	*m*-xylene	rt	TfOH (0.1)	24	24	83
16	*m*-xylene	rt	TFA (0.2)	2	97	91
17	*m*-xylene	rt	TFA (0.05)	2	98	91
18	*m*-xylene	0 °C	TFA (0.1)	21	99	95
19	*m*-xylene	−10 °C	TFA (0.1)	72	94	95
20 ^c^	*m*-xylene	rt	TFA (0.05)	3	99	90
21 ^d^	*m*-xylene	rt	TFA (0.01)	20	96	89

^a^ Isolated yield; ^b^ Determined by HPLC analysis; ^c^ Catalyst (0.05 equiv) was used; ^d^ Catalyst (0.01 equiv) was used.

In order to identify the scope and limitations of aldehyde substrates, we investigated substituent effects of the branched aromatic aldehydes on the conjugate additions (Table 3). A range of electron-withdrawing substituents such as bromo and fluoro moieties, and electron-donating substituents such as methyl and methoxy groups on the aromatic ring of branched aldehydes **12b**–**g** provided the corresponding adducts in excellent yields with good enantioselectivities (83%–92% ee) (entries 2–7). The additions of branched aldehydes possessing a naphthalene motif, **12h** and **12i**, to vinyl sulfone **11** proceeded smoothly in the presence of a catalytic amount of **6** to afford the corresponding adducts **13h** and **13i** in excellent yields with 92% ee, respectively (entries 8 and 9). Interestingly, 2-methoxy-2-phenylacetaldehyde (**12j**) was also applicable and gave the corresponding adduct **13j** in high yield, albeit with reduced enantioselectivity (entry 10). In addition, **6** promoted the reaction of *N*-Boc α-aminophenylacetaldehyde (**12k**) with **11** to yield the corresponding adduct **13k** in 68% yield with 60% ee (entry 11).

**Table 3:** Conjugate additions using organocatalyst **6**. 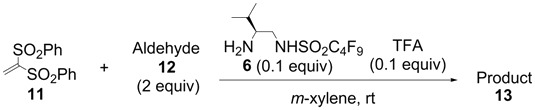

Entry	Aldehyde	Product	Time (h)	Yield ^a^ (%)	ee ^b^ (%)
1	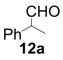	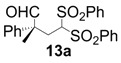	2	95	93
2	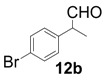	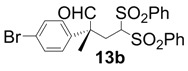	3	97	89
3	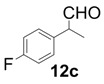	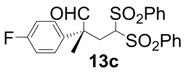	2	95	91
4	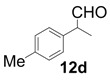	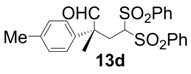	2	99	92
5	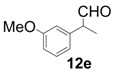	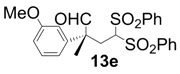	4	99	92
6	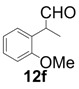	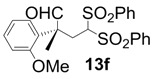	10	98	83
7	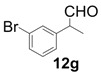	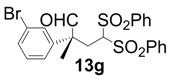	4	99	91
8	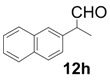	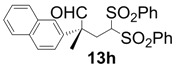	3	99	92
9	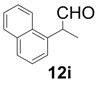	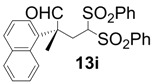	4	97	92
10	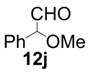	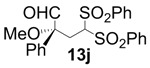	77	88	68
11	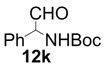	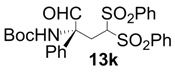	6	68	60

^a^ Isolated yield; ^b^ Determined by HPLC analysis.

Based on the optimal conditions for conjugate additions using **6**, the reaction conditions were optimized for the enantioselective conjugate additions using **7** (Table 4). 1,2-Dichloroethane was the most suitable solvent among those examined in the presence of 0.1 equiv of TFA at room temperature. The reaction in 1,2-dichloroethane provided high yield and enantioselectivity (entry 8). It should be noted that **7** can promote the conjugate additions in brine because the perfluoroalkyl chain of **7** functions as the hydrophobic reaction field in water as described in our previous report [42,43].

**Table 4:** Optimization of reaction conditions using organocatalyst **7**. 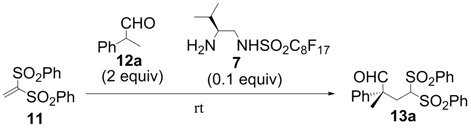

Entry	Solvent	Additive (Equiv)	Time (h)	Yield ^a^ (%)	ee ^b^ (%)
1	toluene	none	24	85	83
2	toluene	TFA (0.1)	2	99	89
3	CH_2_Cl_2_	TFA (0.1)	2	90	91
4	hexane	TFA (0.1)	2	91	86
5	Et_2_O	TFA (0.1)	2	85	89
6	brine	TFA (0.1)	24	68	78
7	CHCl_3_	TFA (0.1)	2	100	91
8	ClCH_2_CH_2_Cl	TFA (0.1)	2	87	92
9	*m*-xylene	TFA (0.1)	2	84	91

^a^ Isolated yield; ^b^ Determined by HPLC analysis.

The generality and substrate scope were probed for the optimal conditions (Table 5). The tendency of reactivities using **7** was quite similar to that using **6**; however, aldehydes **12e**, **12i**, and **12j** were poor substrates and gave low to moderate yields (entries 5, 9, and 10). Interestingly, the stereoselectivity with **12g** was improved up to 94% ee (entry 7). In addition, the yield in the reaction with **12k** was improved up to 100% yield (entry 11).

The recyclability of **7** was evaluated. After use of **7** in the conjugate addition of **12a** to **11** under the optimal conditions, it was readily recovered by the FSPE technique using fluorous silica gel. Furthermore, the recovered catalyst **7** can be reused without further purification, and its catalytic activity was retained for the first reuse. Unfortunately, the catalytic activity of the recovered catalyst **7** decreased significantly for the second reuse.

**Scheme 3:**
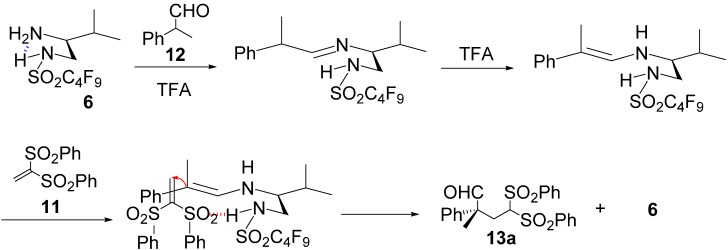
Plausible mechanism and transition state model of reaction.

We infer that the conjugate additions of aldehydes **12** to vinyl sulfone **11** using **6** or **7** proceed via a plausible transition state (Scheme 3) based on the stereochemistry of addition products **13a**–**i**. The primary amino group of **6** or **7** condenses with aldehydes **12** to generate the corresponding imine intermediate. The imine intermediate is subsequently isomerized to the *E*-enamine intermediate because of the resonance stabilizing effect of the aromatic ring. Then, the acidic proton of the sulfonamide group, which coordinates intramolecularly to nitrogen in the enamine transition state, successfully interacts with the oxygen of vinyl sulfone to control the approach direction of vinyl sulfone to the *Re* face of the enamine intermediate. This ultimately affords the corresponding addition products with high stereoselectivities. We believe that the acidity of **6** and **7** is enhanced by the powerful electron-withdrawing effect of the perfluoroalkyl chains, enabling strong coordination to vinyl sulfone and stabilizing the rigid transition states during conjugate additions. Moreover, the addition of TFA to the conjugate additions might accelerate the formation of the imine and enamine intermediates as well as reinforce the rigid transition state of the conjugate additions.

**Table 5:** Conjugate additions using organocatalyst **7**. 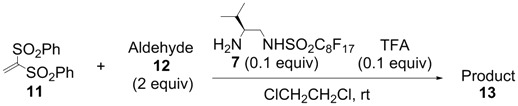

Entry	Aldehyde	Product	Time (h)	Yield ^a^ (%)	ee ^b^ (%)
1	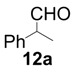	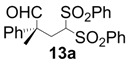	2	87	92
2	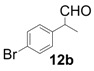	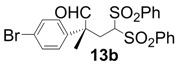	4	90	90
3	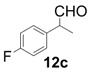	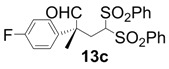	6	100	92
4	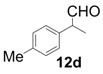	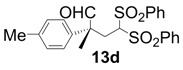	2	92	82
5	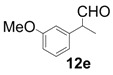	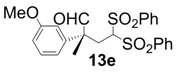	3	13	80
6	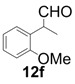	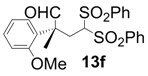	5	100	83
7	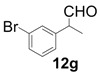	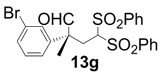	4	81	94
8	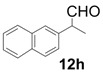	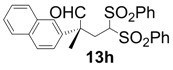	3	76	92
9	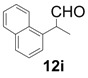	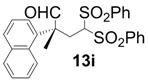	24	45	89
10	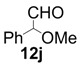	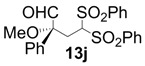	24	64	68
11	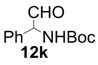	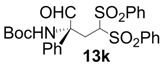	6	100	64

^a^ Isolated yield; ^b^ Determined by HPLC analysis.

## 3. Experimental

### 3.1. General

^1^H-NMR and ^13^C-NMR spectra were measured with a JEOL AL 400 spectrometer (400 MHz for ^1^H-NMR and 100 MHz for ^13^C-NMR), or JEOL ECA-500 spectrometer (500 MHz for ^1^H-NMR and 125 MHz for ^13^C-NMR). The chemical shifts are expressed in ppm downfield from tetramethylsilane (δ = 0.00) as an internal standard. For thin layer chromatographic (TLC) analyses, Merck precoated TLC plates (silica gel 60 F_254_, Art 5715) were used. The products were isolated by flash column chromatography on silica gel (Kanto Chemical, Tokyo, Japan, silica gel 60N, spherical, neutral, 40–50 µm).

### 3.2. Preparation of Organocatalyst **6**

*(S)-tert-Butyl 3-methyl-1-(perfluorobutanesulfonamido)butan-2-ylcarbamate* (**9**). To a solution of (*S*)-*tert*-butyl 1-amino-3-methylbutan-2-ylcarbamate (8, 300 mg, 1.48 mmol) [50] in dry CH_2_Cl_2_ (5 mL) was added triethylamine (0.46 mL, 3.06 mmol) at room temperature under an argon atmosphere. After stirring for 5 min, perfluorobutanesulfonyl fluoride (0.87 mL, 4.45 mmol) was added to the reaction mixture at 0 °C. After stirring for 1 h at 0 °C, the reaction mixture was additionally stirred for 45 h at room temperature. The reaction mixture was added to water and extracted three times with EtOAc. The EtOAc layers were combined, washed with brine, dried over anhydrous MgSO_4_, and evaporated. The residue was purified by flash column chromatography on silica gel with a 7:1 mixture of hexane and EtOAc to give the pure **9** (566 mg, 79%) as a colorless powder. Mp = 74–75 °C; 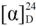 = −5.4° (c = 0.62 in MeOH); ^1^H-NMR (400 MHz, CD_3_OD): δ = 0.81 (d, *J* = 6.8 Hz, 3H), 0.85 (d, *J* = 6.8 Hz, 3H), 1.35 (s, 9H), 1.65–1.71 (m, 1H), 3.08 (dd, *J* = 8.1, 13.5 Hz, 1H), 3.28 (dd, *J* = 4.5, 13.5 Hz, 1H), 3.31–3.37 (m, 1H); ^13^C-NMR (125 MHz, CD_3_OD): δ = 18.2, 19.9, 28.8, 31.0, 47.1, 57.6, 80.2, 110.2–121.0 (complex signals of –CF_2_ and –CF_3_), 158.5; HRMS (ESI-TOF): calcd for C_14_H_21_F_9_N_2_O_4_SNa (M+Na)^+^: 507.0976, Found: 507.0991.

*(S)-N-(2-Amino-3-methylbutyl)-perfluorobutanesulfonamide* (**6**). To a solution of **9** (300 mg, 0.619 mmol) in EtOAc (2.5 mL) was added a 4 M solution of hydrochloric acid in EtOAc (2.5 mL) at 0 °C. After stirring for 2.5 h at room temperature, the reaction mixture was evaporated. The residue was added to saturated aqueous NaHCO_3_ and extracted three times with EtOAc. The EtOAc layers were combined, washed with brine, dried over anhydrous MgSO_4_, and evaporated. The residue was purified by flash column chromatography on silica gel with a 20:1 mixture of CHCl_3_ and MeOH to give the pure **6** (214 mg, 90%) as a colorless powder. Mp = 134–136 °C; 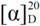 = +7.9° (c = 1.01 in MeOH); ^1^H-NMR (500 MHz, CD_3_OD): δ = 1.01 (d, *J* = 6.9 Hz, 3H), 1.02 (d, *J* = 6.9 Hz, 3H), 1.90–1.97 (m, 1H), 2.82–2.86 (m, 1H), 3.14 (dd, *J* = 8.6, 13.1 Hz, 1H), 3.41 (dd, *J* = 3.5, 13.1 Hz, 1H); ^13^C-NMR (125 MHz, CD_3_OD): δ = 18.9, 19.0, 30.1, 47.4, 60.7, 110.2–120.4 (complex signals of –CF_2_ and –CF_3_); Anal. Calcd for C_9_H_13_F_9_N_2_O_2_S: C, 28.13; H, 3.41; N, 7.29. Found: C, 28.07; H, 3.39; N, 7.26.

### 3.3. Preparation of Organocatalyst **7**

*(S)-tert-Butyl 3-methyl-1-(perfluorooctanesulfonamido)butan-2-ylcarbamate* (**10**). To a solution of (*S*)-*tert*-butyl 1-amino-3-methylbutan-2-ylcarbamate (**8**) [50] (385 mg, 1.90 mmol) in dry CH_2_Cl_2_ (20 mL) was added triethylamine (0.80 mL, 5.71 mmol) at room temperature under an argon atmosphere. After stirring for 5 min, perfluorooctanesulfonyl fluoride (1.57 mL, 5.71 mmol) was added to the reaction mixture at 0 °C. After stirring for 2 h at 0 °C, the reaction mixture was additionally stirred for 90 h at room temperature. The reaction mixture was added to water and extracted three times with EtOAc. The EtOAc layers were combined, washed with brine, dried over anhydrous MgSO_4_, and evaporated. The residue was purified by flash column chromatography on silica gel with a 6:1 mixture of hexane and EtOAc to give the pure **10** (602 mg, 46%) as a pale yellow oil. 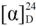 = −4.2° (c = 1.28 in MeOH); ^1^H-NMR (500 MHz, CDCl_3_): δ = 0.95 (d, *J* = 7.4 Hz, 3H), 0.97 (d, *J* = 6.8 Hz, 3H), 1.44 (s, 9H), 1.80–1.85 (m, 1H), 3.25 (m, 1H), 3.46 (brd, *J* = 12.6 Hz, 1H), 3.55 (m, 1H), 4.67 (brd, *J* = 8.0 Hz, 1H), 7.13 (brs, 1H); ^13^C-NMR (125 MHz, CDCl_3_): δ = 18.0, 19.2, 28.2, 30.1, 48.4, 55.7, 80.8, 108.0–113.0 (complex signals of –CF_2_ and –CF_3_), 157.6; HRMS (ESI-TOF): calcd for C_18_H_21_F_17_N_2_O_4_SNa (M+Na)^+^: 707.0848, Found: 707.0873.

*(S)-N-(2-Amino-3-methylbutyl)-perfluorooctanesulfonamide* (**7**). To a solution of **10** (570 mg, 0.833 mmol) in EtOAc (3.5 mL) was added a 4M solution of hydrochloric acid in EtOAc (3.5 mL) at 0 °C. After stirring for 2 h at room temperature, the reaction mixture was evaporated. The residue was added to saturated aqueous NaHCO_3_ and extracted three times with EtOAc. The EtOAc layers were combined, washed with brine, dried over anhydrous MgSO_4_, and evaporated. The residue was purified by flash column chromatography on silica gel with a 20:1 mixture of CHCl_3_ and MeOH to give the pure **7** (444 mg, 91%) as a colorless powder. Mp = 144–145 °C; 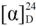 = + 6.9° (c = 1.01 in MeOH); ^1^H-NMR (500 MHz, CD_3_OD): δ = 1.01 (d, *J* = 6.8 Hz, 3H), 1.03 (d, *J* = 6.8 Hz, 3H), 1.90–1.97 (m, 1H), 2.82–2.86 (m, 1H), 3.15 (dd, *J* = 8.5, 13.1 Hz, 1H), 3.41 (dd, *J* = 4.0, 13.1 Hz, 1H); ^13^C-NMR (125 MHz, CD_3_OD): δ = 18.9, 19.0, 30.1, 47.5, 60.7, 109.7–121.5 (complex signals of –CF_2_ and –CF_3_); Anal. Calcd for C_13_H_13_F_17_N_2_O_2_S: C, 26.72; H, 2.24; N, 4.79. Found: C, 26.75; H, 2.41; N, 4.86.

### 3.4. Typical Procedure for Michael Addition (Table 3)

A typical procedure of the Michael additions using **6** is as follows: To a solution of **11** (30.8 mg, 0.100 mmol) and organocatalyst **6** (3.8 mg, 0.010 mmol) in *m*-xylene (1.0 mL) was added 2-phenylpropanal (26.8 µL, 0.200 mmol) and trifluororacetic acid (0.7 µL, 0.010 mmol) at room temperature. After stirring at room temperature for 2 h, the reaction mixture was directly purified by flash column chromatography on silica gel with a 3:1 mixture of hexane and EtOAc to afford the pure **13a** (42.0 mg, 95%) as a colorless powder. All the Michael addition products **13** in the paper are known compounds that exhibited spectroscopic data identical to those reported in the literature [36,37].

*(R)-2-Methyl-2-phenyl-4,4-bis(phenylsulfonyl)butanal* (**13a**). 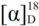 = −25.6° (c = 1.00, CHCl_3_); 95% ee; enantiomeric excess was determined by HPLC with Chiralpak AS-H column (hexane/2-propanol = 70:30), flow rate = 1.0 mL/min; λ = 220 nm; t_major_ = 21.7 min, t_minor_ = 25.9 min.

*(R)-2-(4-Bromophenyl)-2-methyl-4,4-bis(phenylsulfonyl)butanal* (**13b**). 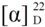 = −15.2° (c = 1.00, CHCl_3_); 89% ee; enantiomeric excess was determined by HPLC with Chiralpak AS-H column (hexane/2-propanol = 70:30), flow rate = 1.0 mL/min; λ = 220 nm; t_major_ = 27.1 min, t_minor_ = 38.5 min.

*(R)-2-(4-Fluorophenyl)-2-methyl-4,4-bis(phenylsulfonyl)butanal* (**13c**). 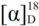 = +23.5° (c = 1.00, CHCl_3_); 91% ee; enantiomeric excess was determined by HPLC with Chiralpak AS-H column (hexane/2-propanol = 70:30), flow rate = 1.0 mL/min; λ = 220 nm; t_major_ = 25.5 min, t_minor_ = 32.1 min.

*(R)-2-Methyl-4,4-bis(phenylsulfonyl)-2-p-tolylbutanal* (**13d**). 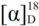 = +25.4° (c = 1.00, CHCl_3_); 92% ee; enantiomeric excess was determined by HPLC with Chiralpak AS-H column (hexane/2-propanol = 70:30), flow rate = 1.0 mL/min; λ = 220 nm; t_major_ = 21.3 min, t_minor_ = 29.7 min.

*(R)-2-(3-Methoxyphenyl)-2-methyl-4,4-bis(phenylsulfonyl)butanal* (**13e**). 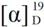 = +10.6° (c = 1.00, CHCl_3_); 92% ee; enantiomeric excess was determined by HPLC with Chiralcel AD-H column (hexane/2-propanol = 80:20), flow rate = 1.0 mL/min; λ = 220 nm; t_major_ = 27.4 min, t_minor_ = 38.6 min.

*(R)-2-(2-Methoxyphenyl)-2-methyl-4,4-bis(phenylsulfonyl)butanal* (**13f**). 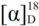 = −70.8° (c = 1.00, CHCl_3_); 83% ee; enantiomeric excess was determined by HPLC with Chiralcel AD-H column (hexane/2-propanol = 80:20), flow rate = 1.0 mL/min; λ = 220 nm; t_major_ = 18.8 min, t_minor_ = 25.9 min.

*(R)-2-(3-Bromophenyl)-2-methyl-4,4-bis(phenylsulfonyl)butanal* (**13g**). 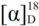 = −71.7° (c = 1.00, CHCl_3_); 91% ee; enantiomeric excess was determined by HPLC with Chiralpak AS-H column (hexane/2-propanol = 80:20), flow rate = 1.0 mL/min; λ = 220 nm; t_major_ = 41.3 min, t_minor_ = 47.1 min.

*(R)-2-Methyl-2-(naphthalen-2-yl)-4,4-bis(phenylsulfonyl)butanal* (**13h**). 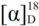 = +13.0° (c = 1.00, CHCl_3_); 92% ee; enantiomeric excess was determined by HPLC with Chiralpak AS-H column (hexane/2-propanol = 70:30), flow rate = 1.0 mL/min; λ = 220 nm; t_major_ = 32.9 min, t_minor_ = 39.1 min.

*(R)-2-Methyl-2-(naphthalen-1-yl)-4,4-bis(phenylsulfonyl)butanal* (**13i**). 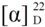 = −31.8° (c = 1.00, CHCl_3_); 92% ee; enantiomeric excess was determined by HPLC with Chiralcel AD-H column (hexane/2-propanol = 70:30), flow rate = 1.0 mL/min; λ = 220 nm; t_major_ = 16.7 min, t_minor_ = 22.4 min.

*(R)-2-Methoxy-2-phenyl-4,4-bis(phenylsulfonyl)butanal* (**13j**). 68% ee; enantiomeric excess was determined by HPLC with Chiralcel AD-H column (hexane/2-propanol = 5:1), flow rate = 1.0 mL/min; λ = 220 nm; t_major_ = 37.1 min, t_minor_ = 44.5 min.

*(R)-tert-Butyl 1-oxo-2-phenyl-4,4-bis(phenylsulfonyl)butan-2-ylcarbamate* (**13k**). 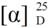 = +10.0° (c = 1.00, CHCl_3_) 64% ee; enantiomeric excess was determined by HPLC with Chiralcel OD-H column (hexane/2-propanol = 90:10), flow rate = 0.5 mL/min; λ = 220 nm; t_major_ = 19.9 min, t_minor_ = 22.2 min.

## 4. Conclusions

Novel organocatalysts **6** and **7** can easily be prepared from l-valine, an inexpensive and commercially available natural amino acid. Organocatalysts **6** and **7**, which are simple β-aminosulfonamides with only one stereogenic center, efficiently catalyze the conjugate additions of various branched aldehydes to vinyl sulfone **11** with a short reaction time at room temperature to give the corresponding addition products possessing all-carbon quaternary stereocenters with high enantioselectivities. The excellent performance is probably due to the carbon skeleton of l-valine and the electron-withdrawing effect of the perfluoroalkyl groups on **6** and **7**. Moreover, fluorous organocatalyst **7** bearing a perfluorooctyl group was readily recovered by simple solid phase extraction using fluorous silica gel and was immediately reusable without further purification for the first cycle. Further application of these organocatalysts in the synthesis of bioactive compounds is currently being investigated in our laboratory.
